# Results of the initial in-brace correction-effect

**DOI:** 10.1186/1748-7161-9-S1-O32

**Published:** 2014-12-04

**Authors:** Kay Steffan, Hans-Jörg Heinen

**Affiliations:** 1 Asklepios Katharina Schroth Klinik, Bad Sobernheim, Germany; 2 Sanomed, Bad Sobernheim, Germany

## Background

Brace treatment for scoliosis is effective. The short term in-brace correction of the Cobb angle is associated with end results and success of bracing. Irrespective of the factors which determine the correction the measurement of the Cobb angle is a possibility to evaluate the brace quality.

We have investigated the braces from one orthotist over a fixed period of time and have described the connection between age, initial Cobb angle and the achieved correction effect.

## Methods

Study design: retrospective. Population: n713 patients diagnosed as having juvenile and adolescent idopathic scoliosis . We differentiated between 1085 curvatures and assessed the changes. Required was a complete ap-y-ray film without brace not older than 6 months prior to the start of the brace treatment and the second x-ray film 2 month after the brace fitting.

The correction effect was described by the difference between the Cobb angle of the 2 x-ray films and expressed as a percentage. Groups were matched regarding Cobb angle and age.

## Results

Median reported correction effect for all 1085 curvatures was 65.4%.

Patients with Cobb < 25° the mean correction was 68.91%, Cobb 26° to 40° mean correction 38.9%, Cobb > 41° correction 28.42%.

## Age groups

< 10yr mean effect 87.56%, N. 57

Cobb < 25° 95.97%, Cobb 26° to 40° 63.12%, Cobb > 40° 34.72%

10 -12yr mean effect 58.83%, N. 100

Cobb < 25° 74.95%, Cobb 26° to 40° 41.79%, Cobb > 40° 31.67%

13-14yr mean effect 50,2%, N. 238

Cobb < 25° 67.25%, Cobb 26° to 40° 38.91%, Cobb > 40° 28.69%

> 14yr mean effect 42.17%, N. 318

Cobb >25° 57.47%, Cobb 26° to 40° 36.58%, Cobb > 40° 27.78%

## Conclusions

The achieved corrections show what we have already expected that younger and less matured patients have the best results. As maturity and the Cobb angle increase the correction effect was reduced by the more structural components of the curvatures.

However the mean correction effect of 65.4% seems to be encouraging. For us the results make clear that to start with bracing early even with Cobb angles below 25° seems to be a sufficient decision within the conservative scoliosis treatment.

